# Ezrin phosphorylation on tyrosine 477 regulates invasion and metastasis of breast cancer cells

**DOI:** 10.1186/1471-2407-12-82

**Published:** 2012-03-07

**Authors:** Hannah Mak, Alexandra Naba, Sonal Varma, Colleen Schick, Andrew Day, Sandip K SenGupta, Monique Arpin, Bruce E Elliott

**Affiliations:** 1 Division of Cancer Biology and Genetics, Cancer Research Institute, Queen's University, Kingston, ON, K7L 3N6, Canada; 2 Laboratoire de Morphogenèse et Signalisation Cellulaires, UMR (Unité Mixte de Recherche) 144 CNRS (Centre National de la Recherche Scientifique), Institut Curie, Paris, France; 3 Department of Pathology and Molecular Medicine, Queen's University, Kingston, ON, K7L3N6, Canada; 4 Koch Institute for Integrative Cancer Research, Massachusetts Institute of Technology, Cambridge, MA 02139, USA

## Abstract

**Background:**

The membrane cytoskeletal crosslinker, ezrin, a member of the ERM family of proteins, is frequently over-expressed in human breast cancers, and is required for motility and invasion of epithelial cells. Our group previously showed that ezrin acts co-operatively with the non-receptor tyrosine kinase, Src, in deregulation of cell-cell contacts and scattering of epithelial cells. In particular, ezrin phosphorylation on Y477 by Src is specific to ezrin within the ERM family, and is required for HGF-induced scattering of epithelial cells. We therefore sought to examine the role of Y477 phosphorylation in ezrin on tumor progression.

**Methods:**

Using a highly metastatic mouse mammary carcinoma cell line (AC2M2), we tested the effect of over-expressing a non-phosphorylatable form of ezrin (Y477F) on invasive colony growth in 3-dimensional Matrigel cultures, and on local invasion and metastasis in an orthotopic engraftment model.

**Results:**

AC2M2 cells over-expressing Y477F ezrin exhibited delayed migration *in vitro*, and cohesive round colonies in 3-dimensional Matrigel cultures, compared to control cells that formed invasive colonies with branching chains of cells and numerous actin-rich protrusions. Moreover, over-expression of Y477F ezrin inhibits local tumor invasion *in vivo*. Whereas orthotopically injected wild type AC2M2 tumor cells were found to infiltrate into the abdominal wall and visceral organs within three weeks, tumors expressing Y477F ezrin remained circumscribed, with little invasion into the surrounding stroma and abdominal wall. Additionally, Y477F ezrin reduces the number of lung metastatic lesions.

**Conclusions:**

Our study implicates a role of Y477 ezrin, which is phosphorylated by Src, in regulating local invasion and metastasis of breast carcinoma cells, and provides a clinically relevant model for assessing the Src/ezrin pathway as a potential prognostic/predictive marker or treatment target for invasive human breast cancer.

## Background

Ezrin is a member of the ezrin-radixin-moesin (ERM) family that functions as a cytoskeletal plasma membrane crosslinker [1,2]. Ezrin is required for epithelial cell integrity and participates in several actin-based functions such as organization of the apical surface of epithelial cells [3,4], cell adhesion [5,6], cell motility and morphogenesis [3,7]. ERM proteins are negatively regulated by an intramolecular interaction between the N-terminal and C-terminal domains, which masks the actin and membrane binding sites [1]. Sequential binding of PIP2 and phosphorylation of a conserved threonine residue (T567) are required for ezrin activation and membrane-cytoskeleton linker function [8].

Ezrin also plays an important role in tumor progression. Gene [9,10] and protein [11-17] expression profiling have revealed a marked increase in ezrin expression in a variety of human and rodent cancers compared to non-malignant tissue counterparts. Moreover, increased cytoplasmic expression of ezrin is frequently associated with dedifferentiation, invasiveness, and poor prognosis in human breast cancers; compared to membranous apical expression in non-malignant epithelial tissues [18]. Furthermore, suppression of ezrin function using shRNA and dominant negative ezrin mutants abrogates invasion, early metastatic survival, as well as lung metastases in osteosarcoma, rhabdomyosarcoma [9,19], and breast carcinoma [20-22] cell lines. In addition, ectopic expression of the suppressor gene, RhoBTB2, causes dephosphorylation of ezrin and inhibits migration and metastasis of breast carcinoma cells [23].

Ezrin is a substrate of the non-receptor tyrosine kinase, Src [2,5,24]. Src expression and activation are frequently up-regulated in breast cancer, and Src is typically recruited to both focal adhesions and cell-cell contacts. It is required for anchorage-independent growth, cell migration and invasion [25,26]. Activated pY419 Src has been found to be associated with decreased disease-specific survival in human breast cancer patients [27-29]. Over-expression of an activated form of Src in transgenic mice induces mammary hyperplasias, which infrequently progress to tumors [30]. Furthermore, Src kinase is required for polyoma middle T-induced mammary tumorigenesis in transgenic mice [31]. Using a mouse breast carcinoma cell line (SP1), we have shown previously that c-Src kinase activity is required for HGF-dependent cell motility and anchorage-independent growth [32]. Collectively, these findings indicate that c-Src kinase activity is an important requirement for mammary tumorigenesis.

We and others have recently identified a co-operative role of Src and ezrin in regulating disruption of cadherin-based cell-cell contacts [33], cell spreading and cell morphogenesis [5] in epithelial cells. Furthermore, Src/ezrin co-operativity is regulated through phosphorylation of specific tyrosines (Y145 and Y447) on ezrin by Src [5,24]. Expression of Y145F or Y477F ezrin, mutants that cannot be phosphorylated by Src, delays HGF-induced cell spreading of epithelial cells on fibronectin substratum. Naba *et al. *[2,6] further showed that interaction of Fes kinase with pY477 of ezrin promotes HGF-induced scattering of epithelial cells, suggesting the ezrin/Fes interaction is linked to epithelial-mesenchymal transition [2,6]. In addition, Heiska *et al. *[34] have recently shown that Y477 ezrin is required for growth and invasion of Src-transformed fibroblasts in 3-dimenional (3D) matrix cultures. However the role of ezrin phosphorylation on tyrosine 477 in tumor progression and metastatic dissemination remains unknown.

Based on the above findings, we hypothesized that phosphorylation of Y477 on ezrin by Src, could regulate the invasive and metastatic potential of mammary carcinoma cells, and thus breast tumorigenesis and progression. Using a model of orthotopic engraftment of a mouse mammary carcinoma tumor cell line (AC2M2) [20], we found that over-expression of a nonphosphorylatable Y477F ezrin mutant markedly decreased local invasion of primary tumor transplants, compared to control vector expressing tumor transplants which rapidly infiltrated into underlying abdominal wall and visceral tissues. In support of these observations, Y477F ezrin-expressing AC2M2 cells formed cohesive round colonies with few cellular extensions in 3-dimensional (3D) Matrigel cultures, compared to control vector expressing cells which showed marked invasion of branching chains of cells with numerous actin-rich protrusions. Altogether, these findings implicate a pivotal regulatory role of the Src phosphorylation site, Y477, on ezrin in invasion and dissemination of breast cancer cells.

## Methods

### Cell lines and tissue culture

The AC2M2 cell line is a lung metastatic variant selected from a breast carcinoma cell line (SP1) following serial intramammary injections of lung metastatic nodules into syngeneic mice, as described previously [35]. The AC2M2 cell line was maintained in Dulbecco's modified Eagle medium (DMEM, Invitrogen/Gibco, Burlington, ON) with 10% fetal bovine serum (FBS) at 37°C.

### Cell transfection

The pCB6 vector containing cDNA coding for Y477F ezrin fused to the VSVG tag was previously described [6]. Transfections with pCB6 alone or containing VSVG tagged Y477F ezrin were carried out with Lipofectamine Plus reagent (Invitrogen/Gibco) in accordance with the recommended protocol. Stable transfectants were selected for G418 resistance (400 μg/ml; Invitrogen:Gibco) and cloned. One pCB6 clone and two Y477F ezrin-expressing clones (A43 and C13) were selected for further study.

### Western blotting

Snap frozen tissues were homogenized in ice cold RIPA buffer containing a cocktail of protease and phosphatase inhibitors (Fisher Scientific, Tornonto, ON). Homogenates were sonicated for 15-20 minutes at 4°C, and centrifuged at 13000× rpm for 10 minutes at 4°C. The supernatant was removed and the pellet was discarded. Cultured cells were rinsed with ice-cold PBS supplemented with 0.1 μmol/l CaCl2 and 0.1 μmol/l MgCl2, and lysed in 2× Laemmli buffer. Protein determination of lysates was performed using a DC protein assay kit (Biorad, Mississauga, ON). In one experiment involving pY477 ezrin western blotting, cells were treated with 0.1 mM pervanadate for 5 min before lysis to inhibit phosphatases [6]. All lysates were resolved on 8% polyacrylamide gels for SDS-PAGE under reducing conditions and transferred to PVDF membranes (Immobilon-P membrane, Fisher Scientific). Membranes were blocked and probed with the indicated primary antibodies and appropriate secondary antibodies. Proteins were visualized using enhanced chemiluminescence reagent (Pierce ECL, Fisher Thermo Scientific, Toronto, ON). Densitometric analysis was used to calculate the ratio of ezrin to γ-tubulin, and the value for each clone was normalized to that of pCB6 cells.

### Wound healing assay

Cells were plated in 12 well tissue culture plates (InVitrogen/Gibco) with complete tissue culture medium for next day confluence, wounded and washed, as described previously [20]. Cell migration was monitored over a 24 h period through a Nikon inverted microscope, and images of the same marked fields were captured at various times. The area of wound closure, relative to t = 0 hours was measured in four independent wound sites per cell line using ImageJ software. Relative cell motility was calculated as the difference between the wound area at t = 0 hours and t = 18 or 24 hours, as indicated. Mean values +/- SD per group were calculated, and statistical significance among groups was assessed using one-way analysis of variance (ANOVA).

### 3D Matrigel cultures

3D Matrigel embedded cultures were set up as previously described by Lee *et al. *[36]. Pre-chilled 8 chamber glass cover slip plates (VWR/BD Biosciences, BD Biosciences, Burlington, ON) were coated with a thin layer (30 μl) of 100% growth factor-reduced Matrigel (VWR/BD Biosciences), and incubated at 37°C for at least 30 min to allow for gelling. AC2M2 clones (7500 cells/well) expressing pCB6 vector alone, or with Y477F ezrin, were harvested, resuspended in 75% Matrigel with complete medium (DMEM/F12 + 5% FBS + 1% P/S + 5 μg/ml Insulin), plated (150 μl/well) onto the coated surface, and allowed to gel for 30 min at 37°C. 5% Matrigel was added to the remaining chilled complete medium, for top up of cultures (200 μl/well) and subsequent feeding every two days. Cultures were monitored over a 12 day period, during which time tumor cells formed clusters from single cells, referred to subsequently as "colonies", as previously described [36,37]. Phase contrast photographs of colonies were taken at various times using an inverted Olympus microscope. The number of colonies per well was counted, and colony forming ability was calculated as the percentage of colonies formed from 7.5 x 103 cells plated per well (average of 3 wells per group). Morphology of colonies was assessed as "invasive" structures with branching chains of cells (extensions) and cellular protrusions, or cohesive "round" colonies with no chains or protrusions. The presence of 5 or more cellular extensions or protrusions per colony was considered positive. At least 300 colonies were counted per group. Statistical significance among groups was determined by one-way ANOVA.

### Cell growth assay

Cell growth *in vitro *was assessed using an MTT (Methylthiazolyldiphenyl-tetrazolium) assay (Sigma-Aldrich, Oakville, Ontario), according to manufacturer's specifications. See Additional file 1: Figure S1 for details.

### Immunofluorescence

Cell colonies from Matrigel cultures were fixed in the chamber wells with a final concentration of 4% paraformaldehyde in PBS for 30 min, permeabilized with 0.5% Triton X100 for 15 min, and blocked for 1.5 h in immunofluroescence (IF) buffer (130 mM NaCl, 7 mM Na2HPO4, 3.5 mM NaH2PO4, 7.7 mM NaN3, 0.1% BSA, 0.2% Triton-X 100, 0.05% Tween 20) with 10% goat serum. Cells were stained for 2.5 h at 4°C with rabbit anti-ezrin and anti-VSVG antibodies (prepared as previously described [38]). Cells were then washed 3 times with IF buffer, incubated for 1 h with Alexa546 anti-rabbit IgG (Invitrogen), washed 3 times with IF buffer, followed by DAPI staining for 15 min, and rinsing with PBS. In some experiments, Alexa488-phalloidin (Invitrogen/Gibco) was used to detect F-actin. Preparations were imaged using a Quorum Wave FX spinning disk confocal microscope (Quorum Technologies, Inc.) in the Queen's Cancer Research Institute and Protein Discovery and Function Facility. 3D reconstruction of deconvoluted image stacks was carried out using Metamorph imaging software (Molecular Devices, Inc., Sunnyvale, CA). Images shown are representative of at least 20 colonies in three experiments.

### Tumor transplantation

In this model, we have previously shown that 100% incidence of primary tumor takes occurs in mice engrafted into the mammary gland with 7.5 × 103 tumor cells, with tumor diameter reaching 1 cm within an average of 27 days. AC2M2 cells were injected (7.5 × 103 cells in 10 μl/mouse) into the mammary fat pad of athymic female nude (nu-/-) mice (Tac: NCRNUM, Taconic Farms), as described previously [20]. Immunodeficient nude mouse recipients were used to minimize the T cell-dependent effects of the immune system on tumor growth and metastasis, and to allow assessment of tumor lesions (e.g, shape, texture, and adhesion to abdominal wall) in real time during early stages of tumor growth. Mice were housed in the Animal Care Facility and procedures were carried out according to the guidelines of the Canadian Council on Animal Care, with the approval of the institutional animal care committee. Control tumors engrafted into the mammary fatpad of nude mice showed a similar pattern of metastatic spread as previously found with the syngeneic engraftment model [20]. Primary tumor growth was monitored and measured with callipers every 2-3 days. Perpendicular dimensions were measured and tumor volume was calculated using the following formula: (a)(b2)/2; where 'a' was the larger of the two measurements. The natural log of the tumor volumes as measured on days 15, 17, 20, and 23 (representing a logarithmic growth period) was compared among the groups by a linear mixed effect model [39], and significance was assessed using a global F-test (SAS Institute Inc., 2008). (See Additional file 2: Figure S2).

Primary tumors were surgically removed at 21 and 23 days (in 2 experiments, respectively), at which time tumor diameters were close to 1 cm and local invasion was observed. Animals were allowed to recover, and were monitored for 20 more days to allow distant metastases to grow and to assess local recurrence, as determined previously [35]. Gross pathology of resected primary tumors, as well as abdominal and thoracic (lung) organs, and inguinal, axillary and iliac lymph nodes removed at autopsy was assessed. Portions of tissues were either snap frozen for biochemistry, or fixed in 4% paraformaldehyde and paraffin embedded (FFPE). Fixed tissue sections (5 μm thickness) were stained with hematoxylin and eosin to assess primary tumors, abdominal and thoracic organs for presence of local invasion and metastases. Six mice were included in each group of two experiments, and results were combined for analysis.

### Scoring and analysis of local invasion and metastasis

Local invasion was defined at the gross pathological level primarily as adhesion to, or invasion of tumor cells into, the adjacent abdominal muscle wall. At the histological level, tumors were classified as non-invasive if a clear circumscribed border between the tumor and the stroma was observed. Tumors that were invasive often displayed tumor extensions into the surrounding fatpad, and spreading into the abdominal wall or draining lymph node. Seeding of tumor cells and invasion into visceral organs was also recorded. Invasion based on any of the above criteria was assessed as a categorical factor (presence or absence), and a two-sided non-parametric Fisher exact test was used to assess statistical significance among control pCB6 and Y477F ezrin-expressing clones.

For metastasis assessment, lungs from tumor bearing mice were processed as described above, and 5 μm serial sections of paraffin embedded tissues were stained with hematoxylin and eosin for histopathological examination. At least two sections from the superficial and middle regions of each mouse lung were examined, and the number of lung nodules per mouse was determined. The proportion of mice with metastases (positive or negative) in each group was tabulated with significance determined by a two-sided Fisher exact test. The frequency of lung lesions (number of lung nodules) for each mouse in each group is shown as a dot plot, with significance determined by a non-parametric Wilcoxon Rank Sum test.

## Results

### Expression of Y477F ezrin mutant attenuates migration of breast carcinoma cells

**Figure 1:**
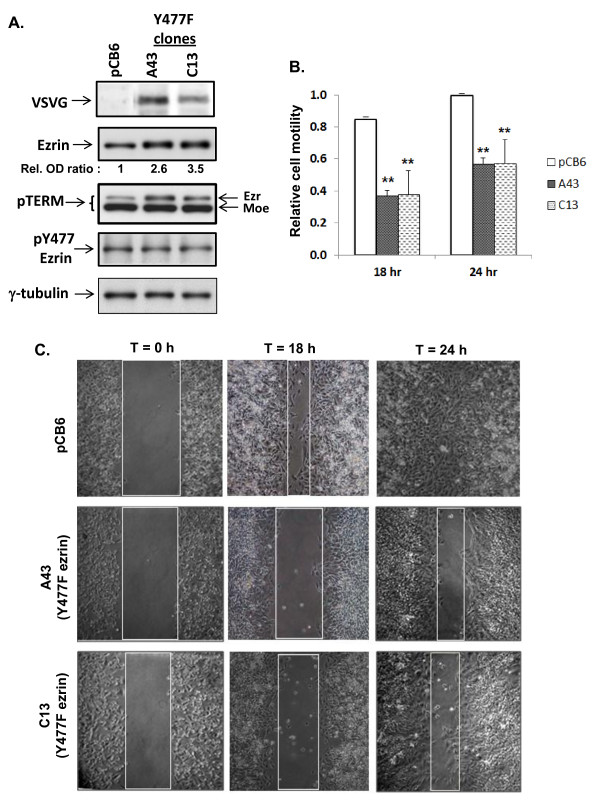
**Effect of Y477F ezrin on cell motility in AC2M2 cells**: **Panel A) **AC2M2 cells transfected with an empty pCB6 vector, or expressing Y477F ezrin, clones (A43 and C13) were lysed in Laemmli buffer, and equal protein amounts were subjected to SDS-PAGE and western blotting with antibodies against VSVG, ezrin, pY477 ezrin, pTERM and γ-tubulin. In the ezrin blot, the ~81 kDa band in pCB6 represents endogenous ezrin, while the bands in A43 and C13 represent both Y477F mutant and endogenous ezrin. Optical density (OD) ratios of ezrin vs γ-tubulin bands show a 2.6-fold and 3.5-fold increase in total ezrin expression of the A43 and C13 clones, respectively, normalized to the pCB6 clone. Ezr, ezrin; moe, moesin. **Panel B) **AC2M2 clones expressing either pCB6 empty vector or Y477F ezrin were grown to confluence in 10% FBS/DMEM. Confluent cells were wounded by scoring and medium was immediately replaced. Spontaneous wound closure at each of four marked wound sites for each cell clone was monitored for up to 24 h by phase contrast microscopy. The histogram shows relative cell motility for each clone calculated as the area of wound closure at 18 h and 24 h compared to T = 0 h. Values represent the mean +/- SD of 4 wound sites per clone in each of two experiments. There was significant reduction in cell motility in Y477F ezrin expressing clones (A43 and C13) at both 18 h and 24 h, as determined by a one-way ANOVA (*p *< 0.001). **Panel C) **Representative fields photographed using phase contrast microscopy (10× objective) at 0 h, 18 h and 24 h are shown. Boxed areas indicate wound area measured at each time point.

Since cell migration is an important early step in tumor invasion, we examined the role of Y477 ezrin on migration function of a highly metastatic carcinoma variant cell line, AC2M2, derived from the mouse mammary tumor cell line, SP1 [35]. SP1 and AC2M2 cells express HGF and activated Met, and spontaneously migrate in a wound healing assay ([40], unpublished data). We generated stable transfectants of AC2M2 cells expressing either a VSVG-tagged Y477F ezrin mutant in a pCB6 eukaryotic expression vector (clones A43 and C13), or an empty vector (control) (Figure 1A). A VSVG-tagged protein corresponding to the 80 kDa ezrin band was detected in both A43 and C13 cells. Total ezrin protein levels were approximately 3-fold higher in A43 and C13 clones compared with the pCB6 clone, indicating overexpression of Y477F-ezrin compared to endogenous protein. Likewise, phosphorylated T567 ezrin (upper band) showed increased expression in Y477F ezrin compared to pCB6 clones, while phosphorylated T568 moesin remained unchanged. Endogenous levels of pY477 were similar among the three cell clones.

AC2M2 cells expressing Y477F ezrin showed a significantly reduced rate of wound closure at 18 h and 24 h, compared with control cells which spontaneously migrate (Figure 1B, C). Less than confluent cultures showed no significant effect of Y477F ezrin on wound closure, indicating that the observed phenotype was dependent on cell density (data not shown). Thus expression of Y477F ezrin attenuates migration of confluent breast carcinoma cells.

### Y477F ezrin mutant inhibits invasion of breast cancer cells in 3D Matrigel culture

**Figure 2:**
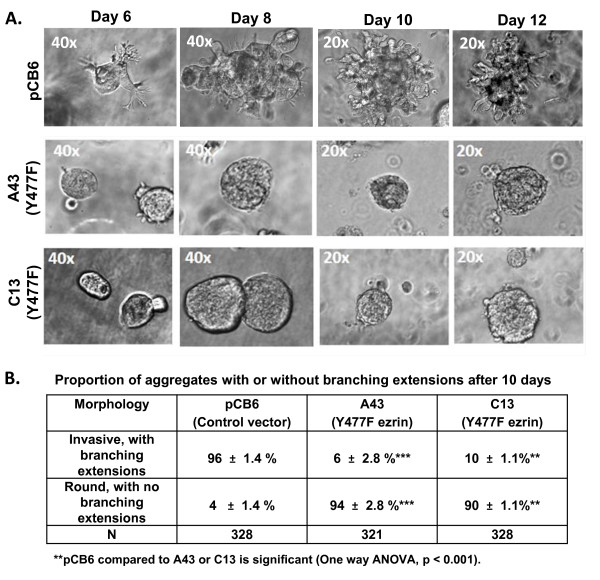
**Effect of Y477F ezrin on morphology of AC2M2 cells in 3D Matrigel cultures**: **Panel A**: AC2M2 cell clones expressing pCB6 empty vector or Y477F ezrin (clones A43 and C13) were cultured in 3D Matrigel. Colony morphology was assessed at the times indicated, using phase contrast microscopy. Objective magnification is shown in each panel. **Panel B: **Table indicates percentages of invasive colonies from each cell clone with branching cellular extensions and protrusions, and of cohesive round colonies with no extensions, respectively. The presence of 5 or more cellular extensions or protrusions per colony was considered positive. Mean percentage of colonies +/- SD (3 wells per group) in each category is shown. N = total number of colonies counted. pCB6 cells formed predominantly invasive colonies with cellular extensions, whereas the majority of Y477F ezrin cells formed round colonies with no extensions. The above differences in colony morphology were significant as determined by one way ANOVA (*p *< 0.001).

Since tumor cell invasion is affected by the 3D matrix milieu [41], we decided to further characterize the Y477F ezrin phenotype in a 3D Matrigel embedded culture system [36]. Interestingly, AC2M2 cells expressing control vector or Y477F ezrin (clones A43 and C13) showed no significant difference in overall cell growth or colony forming ability in 3D Matrigel cultures (Additional file 1: Figure S1). However the morphology of the Y477F ezrin compared to control groups was quite distinct (Figure 2). By 12 days, control cells formed predominantly (91%) colonies with branching chains of cells (extensions) and cellular protrusions, characteristic of a migratory invasive phenotype. In contrast, the majority (92-96%) of Y477F ezrin cells formed colonies exhibiting a cohesive round morphology with very few cellular extensions or protrusions.

**Figure 3:**
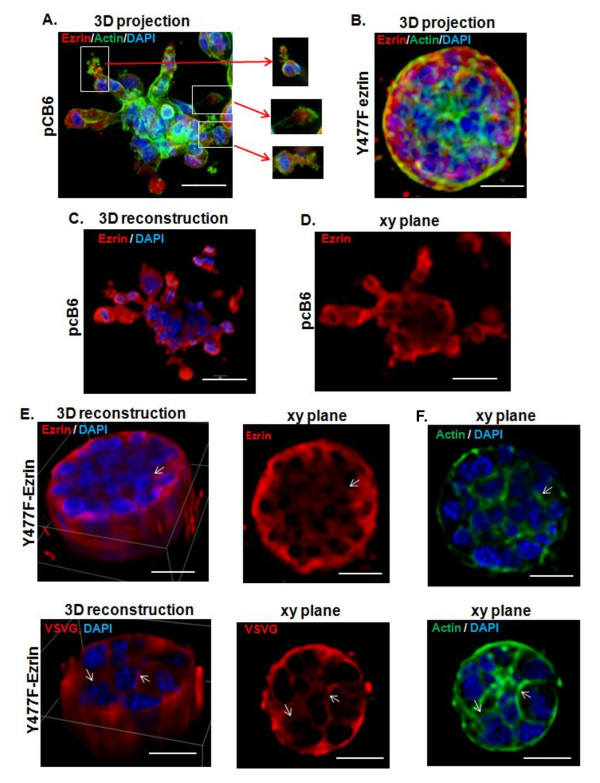
**Cellular localization of ezrin and Y477F ezrin (VSVG tagged) in colonies growing in 3D Matrigel culture**: Colonies formed from AC2M2 cells expressing pCB6 control vector or Y477F ezrin (clone C13) in day 12 embedded Matrigel cultures (see Figure 2) were fixed, and stained for ezrin (red), actin (green, phalloidin), and nuclei (blue, DAPI). Images of stained colonies were acquired using a Quorum Wave FX spinning disk confocal microscope. **Panels A & B) **3D projection images of pCB6 control and Y477F ezrin expressing clones are shown (three colours merged). Inserts show single XY plane of actin-rich cellular protrusions with ezrin localized to the cytoplasmic side in pCB6 colonies. No actin-rich protrusions were evident in Y477F ezrin colonies. **Panel C) **Images of pCB6 colonies were deconvoluted and subjected to 3D reconstruction using Metamorph software. A middle section showed strong localization of ezrin in cellular extensions, compared to weak expression in the central core of colonies. **Panel D**) A middle xy plane of the pCB6 colony shown in **C **confirmed the absence of a hollow lumen. **Panel E**) Deconvoluted images of Y477F ezrin expressing colonies stained for ezrin or VSVG were subjected to 3D reconstruction as above. A middle xy plane showed a partial membranous staining which was strongest in the outer surface of colonies of Y477F ezrin-expressing cells, though some diffuse cytoplasmic staining was also evident. No hollow lumens were present. **Panel F**) XY planes corresponding to ezrin and VSVG staining in **E **show actin structures associated with the plasma membrane particularly at cell junctions. Images are representative of at least 20 colonies examined in each group. Scale bar indicates 50 μm.

To assess the architectural organization of cell colonies in 3D culture, expression and localization of total ezrin, Y477F-ezrin (VSVGtagged)and F-actin were examined using confocal spinning disk microscopy and deconvolution image analysis. 3D projection images of pCB6 cell colonies confirmed the presence of branching chains of cells, characteristics of invasive breast carcinoma cells [37,42] (Figure 3A). Moreover, the distal leading cells showed numerous actin-rich cellular protrusions, characteristics of migratory cells. Ezrin was strongly localized in these actin-rich structures, and weakly expressed in the central region of pCB6 colonies (Figure 3C, D). In contrast, the colonies formed by Y477F ezrin expressing cells exhibited a cohesive round morphology and failed to form chains or protrusions, resulting in an overall smooth surface (Figure 3B, E). In these colonies intense actin staining was evident in the cortical cellular region (Figure 3F). Furthermore, both ezrin and VSVG (Y477F) ezrin showed a partial membranous staining which was strongest in the outer surface of colonies of Y477F ezrin expressing cells, though some diffuse cytoplasmic staining was also evident (Figure 3E). XY plane images through the middle of either pCB6-or Y477F ezrin-expressing colonies revealed no hollow lumens (Figure 3D, E). Thus, the presence of actin-rich cellular protrusions with marked ezrin expression is a distinguishing feature of pCB6-expressing colonies, while the expression of Y477F ezrin inhibited this invasive phenotype.

### Y477F ezrin inhibits local invasion but not growth of primary tumor outgrowths

Our finding that Y477F ezrin attenuates the invasive tumor phenotype in 3D Matrigel cultures *in vitro *prompted us to assess the effect of Y477F ezrin on *in vivo *tumor progression using our highly metastatic AC2M2 carcinoma cell line model [20]. AC2M2 cell clones expressing control vector or Y477F ezrin were engrafted into the mammary gland of recipient mice and primary tumor growth was monitored every 2-3 days. Primary tumors were excised at 21 or 23 days (two experiments), at which time lesions were close to 1 cm in diameter. A VSVG-tagged 80 kDa protein was detected by western blotting on protein extracts from Y477F ezrin expressing tumors, indicating the presence of the mutant ezrin form in the tumor tissues. (Additional file 2: Figure S2A). No significant difference in mean tumor volume per group between pCB6 control and Y477F ezrin-expressing tumors over days 15, 17, 20 and 23 (representing a logarithmic growth period) was observed (overall *p *= 0.57) (Additional file 2: Figure S2B).

**Figure 4:**
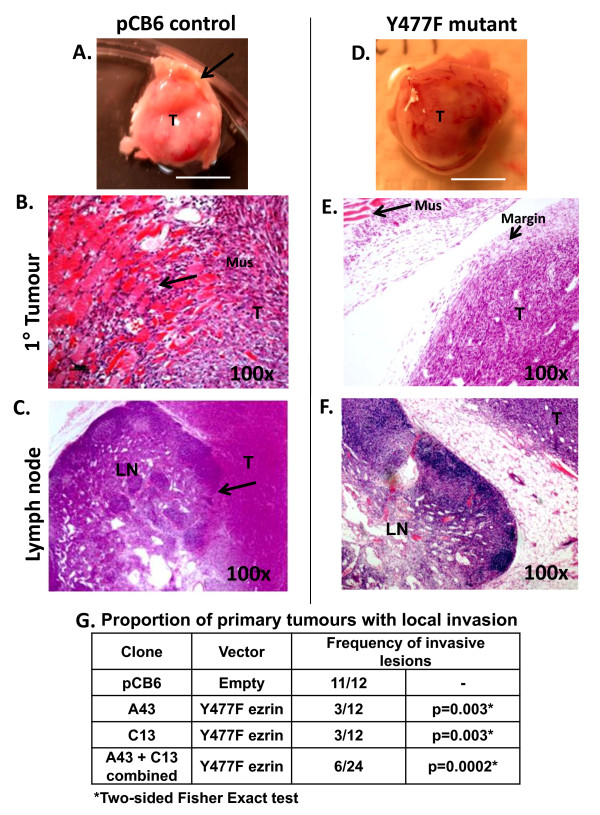
**Effect of the expression of Y477F ezrin on local invasion of AC2M2 breast carcinoma cells**: Two clones of AC2M2 cells expressing Y477F ezrin (A43 and C13) or empty pCB6 vector were engrafted into the mammary fat pad of nude mice. Primary tumors from the mice were excised after 21 or 23 days, and photographs of gross pathology (**Panels A, D**) were taken. Scale bar = 0.5 cm. 5 μm sections of FFPE processed tissues were stained with hematoxylin and eosin for histopathological analysis. Arrows indicate invasion of pCB6 tumor into underlying abdominal muscle wall (**Panels A, B**) or draining lymph node (**Panel C**). In contrast, Y477F ezrin tumors showed no adhesion to abdominal wall (**Panels D, E**) and rarely penetrated into the circumscribed tumor margin (**Panel E**) or adjacent lymph node (**Panel F**). Invasion was assessed categorically (presence or absence), based on combined observations of gross and histopathology. Data were expressed as the proportion of mice with local invasion relative to the total number of mice per group. Significant difference between pCB6 control and Y477F ezrin-expressing clones (A43 and C13) in two pooled experiments is shown (*p* = 0.003, two-sided Fisher Exact test). The overall p value for A43 + C13 clones combined is 0.0002 (**Panel G**). Label with "T" indicates tumor, "Mus" indicates muscle and "LN" indicates lymph node. Image magnification is shown in each panel.

Local invasion and spreading of pCB6 control and Y477F ezrin expressing AC2M2 tumors were characterized based on gross and histopathological assessment of primary lesions excised after 21-23 days (corresponding to Additional file 2: Figure S2B). At this time control tumors are locally invasive but overt metastases in distant organs are not yet visible. On gross pathological examination, the majority (11/12) of pCB6 control tumors were found to invade through the abdominal wall and underlying muscle (Figure 4A), with tumor nodules frequently visible in visceral organs (Additional file 3: Figure S3). Histopathological analysis of excised primary tumor tissues confirmed that the majority of pCB6 tumors had invaded into the surrounding mammary fatpad, underlying abdominal muscle and adjacent lymph node (Figure 4B, C). Seeding of tumor cells in the viscera, disseminating into various abdominal organs via peritoneal spread, was also confirmed (Additional file 3: Figure S3). Variable organ involvement included intestine, pancreas and spleen. In a rare instance, a suggestive perineural invasion was seen in one of the pCB6 control tumors. Thus, extensive local invasion and seeding of visceral organs by pCB6 control tumors was clearly visible. In contrast, few of the Y477F ezrin expressing tumors (6/24, pool of two independent clones) showed local invasion or seeding of visceral organs at the same time point (Figure 4D-F). Moreover, the majority of Y477F ezrin tumors remained circumscribed, with no invasion into surrounding stroma or adhesion to the abdominal wall (*p *= 0.0002, two-sided Fisher Exact test) (Figure 4G). These results show that Y477F ezrin attenuates local invasion of AC2M2 tumor cells following engraftment into the mammary fatpad.

### Y477F ezrin reduces the frequency of lung metastatic lesions in tumor engrafted mice

**Figure 5:**
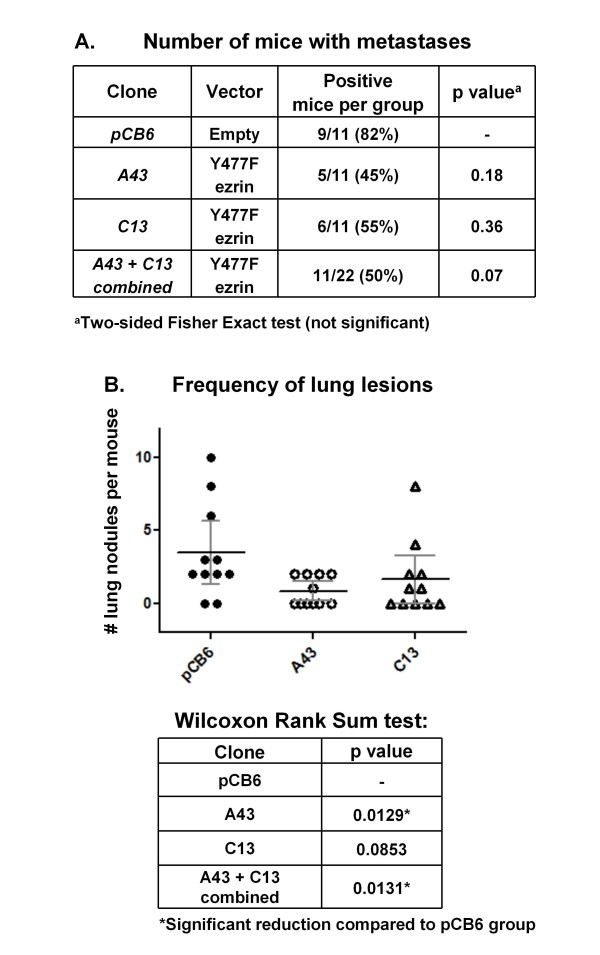
**Effect of the expression of Y477F ezrin on lung metastasis formation**. Mice from which primary tumors were removed (corresponding to Additional file 2: Figure S2) were allowed to survive for 20 additional days to allow overt metastases to form. Serial sections (5 μm) of FFPE processed lung tissues were stained with hematoxylin and eosin for histopathological assessment. At least two sections from the superficial and middle planes, respectively, of each mouse lung were examined and the number of lung nodules per mouse was determined. **Panel A) **The table shows the proportion of mice with metastasis in each group. The incidence of mice with lung metastases from Y477F ezrin tumors (A43 and C13 clones) was reduced, but this difference did not achieve significance, as determined by a two-sided Fisher Exact test. **Panel B) **The number of lung nodules per mouse per group is represented as a dot plot. Statistical significance was determined by a Wilcoxon Rank Sum test. Mean +/- 95% confidence intervals (bars) is shown for each group. A significant reduction in the number of nodules per mouse with clone A43 tumors was observed (*p *= 0.0129). The number of lung nodules in C13 tumors was also reduced, but this value was not significant (*p *= 0.0853), due to one outlier. Analysis of pooled data showed a significant reduction in lung nodules in mice with A43 and C13 tumors combined, compared to mice with pCB6 tumors (*p *= 0.0131).

To assess the effect of Y477F ezrin on distant metastasis, mice were allowed to survive for an additional 20 days following excision of primary tumors expressing pCB6 or Y477F ezrin (clones A43 and C13) to allow outgrowth of lung metastases. The presence of lung metastases was assessed by histological examination of serial tissue sections stained with hematoxylin and eosin, as described in *Materials and Metho*ds (see also Additional Additional file 3: Figure S3G-H). Although there was a trend towards a reduced incidence of lung metastases in mice bearing Y477F ezrin tumors (both A43 and C13 clones), this difference did not achieve significance (*p *= 0.07, Figure 5A). However, when the number of lung nodules per mouse was assessed, the frequency of lung metastatic lesions was significantly reduced in mice with clone A43 tumors (*p *= 0.0129) (Figure 5B). While fewer lesions in mice with clone C13 tumors were also observed, this difference was not significant (*p *= 0.0853) due to one outlier mouse (with 8 nodules). However, analysis of pooled results showed that the frequency of lung lesions from both Y477F ezrin clone tumors (A43 and C13) was significantly reduced, compared to pCB6 tumors (*p *= 0.0131). Thus, ectopic expression of Y477F ezrin significantly reduces the frequency of lung metastatic lesions following orthotopic engraftment of AC2M2 tumor cells into the mammary fatpad.

## Discussion

Our previous demonstration of Src/ezrin co-operativity [33], and the required Y477 phosphorylation of ezrin by Src in HGF-induced scattering of epithelial cells [6], prompted us to examine the role of Y477 ezrin in primary tumor growth, local invasion and metastasis in an *in vivo *model of malignant breast cancer. Y477 is of particular interest since this residue is specific for ezrin and is not present in the two other ERM proteins, radixin or moesin, that are highly homologous to ezrin [2,6]. In this report, we show for the first time that Y477F ezrin attenuates local invasion and distant metastasis of breast carcinoma cells transplanted into the orthotopic site of recipient mice. Moreover, Y477F ezrin promotes formation of round colonies by carcinoma cells embedded in 3D Matrigel culture, compared to formation of invasive colonies by control cells. Thus, phosphorylation of Y477 ezrin plays a key role in local invasion and metastasis from the primary tumor site.

The AC2M2 breast carcinoma cell line expresses elevated Src activity, spontaneously migrates, invades through transwell cultures, and metastasizes to the lung following engraftment into the mammary fat pad [20,32]. An approximate 3-fold over-expression of Y477F ezrin compared to endogenous ezrin was sufficient to block spontaneous migration of AC2M2 cells in a wound healing assay, consistent with a dominant inhibitory effect of the ezrin mutant. Therefore the AC2M2 cell line model is ideally suited for studying regulation of malignant breast tumor progression by phosphorylatable Y477 ezrin.

Our observed invasive colonies of pCB6 cells with cellular extensions and protrusions in 3D Matrigel cultures is similar to the aggressive "stellate" morphology identified by Han *et al. *in certain human breast cancer cell lines (e.g. MDA-MB-231) with basal-like characteristics [42]. In contrast, AC2M2 cells expressing Y477F ezrin formed predominantly round colonies with no cellular extensions or protrusions. However, cell growth and lumen filling was unaffected, indicating a partial reversion of the malignant phenotype that has been described as "cyst-like" by others [37,43].This cyst-like phenotype is similar to the "round" morphology identified by Han *et al. *[42], and has been shown to be associated with less aggressive cancers. Gene signatures corresponding to these morphologic phenotypes were shown to accurately predict clinical outcome in independent datasets of human breast cancers [44].

The presence of numerous actin-rich cellular protrusions, in which ezrin is localized in pCB6 colonies, is consistent with the previously reported localization of ezrin in invasive cancers [45,46]. In contrast, Y477F ezrin colonies showed no actin-rich protrusions, indicating a loss of invasive function; while strong actin staining in the cortical region of cells was evident. Together, these findings are consistent with previous reports that Y477F ezrin promotes increased cell-cell contacts and inhibits HGF-induced cell scattering in kidney epithelial cells [2,6]. Interestingly, pCB6 cells expressed low E-cadherin levels and undetectable N-cadherin, and lacked robust cell-cell adhesion ([20], data not shown). This lack of cadherin engagement and reduced cell-cell contacts is frequently associated with more aggressive human breast cancers such as the basal-like subset [47].

In our *in vivo *mammary tumor progression model, gross pathology and histological analysis showed that the majority (> 90%) of primary control tumors engrafted into mammary fatpads of recipient mice invaded into surrounding fat tissue and underlying abdominal muscle within 21 days post engraftment. In addition, marked seeding and dissemination into visceral organs including intestine, spleen, and pancreas were frequently evident. One example of perineural invasion which occurs in about 10% of human invasive breast cancer cases [48], was also observed. Thus, control tumors displayed a phenotype similar to clinically advanced human breast cancers, where locally invasive primary tumors tend to anchor to the chest wall and invade into underlying muscle tissue [49].

In contrast to control tumors, few (25%) of the Y477F ezrin tumors showed locally invasive characteristics. The majority of Y477F ezrin tumors remained circumscribed, with little invasion into surrounding stroma and abdominal wall. Interestingly, no significant effect of Y477F ezrin on the rate of primary growth of AC2M2 tumors was observed, thus mimicking the effect of this mutant on the growth behaviour of AC2M2 cells in 3D culture. This novel finding suggests that phosphorylation at a single tyrosine residue (Y477) on ezrin plays a critical role in local invasion of tumor cells, while having no detectable effect on primary tumor outgrowth.

While Y477F ezrin was found to have a marked attenuating effect on local invasion during early phases of tumor growth, a reduction in the proportion of mice with distant metastasis was also observed, though this effect failed to reach significance. However, a significant reduction in the frequency of lung lesions, an indication of metastatic tumor load, was observed in Y477F ezrin clones compared to pCB6 control cells. These findings show that Y477F ezrin significantly reduces metastatic efficiency, perhaps due to reduced rate of release of tumor cells from the primary lesion, reduced extravasation, or decreased efficiency in establishing colonies in distant organs. The presence of residual metastases in a small proportion of mice bearing Y477F ezrin-expressing tumors could indicate alternate routes of dissemination independent of Y477 ezrin function.

The mechanism by which pY477 ezrin regulates tumor invasion in AC2M2 cells is not known. However, recent evidence shows that Src interaction via phosphorylation of Y477 ezrin regulates HGF-induced scattering and tubule formation in porcine kidney epithelial cells grown in 3D collagen gels (data not shown, and [6]). In this model, pY477 of ezrin interacts with Fes kinase, causing its activation and localization to cell-cell contacts, and phosphorylation by Fes of junctional proteins--a necessary step for cell scattering. We can hypothesize that in our model, the formation of round colonies observed in 3D matrigel when Y477F ezrin is expressed could be due to the lack of interaction with binding partners such as Fes. Similarly, the absence of actin-rich protrusions in cells expressing Y477F ezrin could be due to the lack of interaction between ezrin and currently unidentified partners involved in actin cytoskeleton reorganization. Moreover, pY477 of ezrin may act via pathways that connect Src to receptor tyrosine kinases such as Met, resulting in invasive properties of tumor cells [2]. Consistent with this concept is our recent demonstration of Src/ezrin co-operativity in breast epithelial cells through increased Met activation and degradation of matrix substratum, characteristic of the invasive phenotype [50]. Further studies are required to elucidate the role of Src/ezrin pathway and Y477 ezrin phosphorylation in the invasion process.

## Conclusions

In the present study, we show for the first time that a single tyrosine-to-phenylalanine substitution (Y477F) in ezrin markedly attenuates local invasion and distant metastasis of breast cancer cells from the primary tumor site. Phosphorylation of Y477 ezrin by Src is required for HGF-induced scattering [6]. Y477 is also unique to ezrin, thus distinguishing the observed Src/ezrin synergy effect from other ERM protein interactions. Therefore, our study implicates a unique role of Src/ezrin in regulating local invasion of breast carcinoma cells, and provides a clinically relevant model for assessing the potential of this pathway as a prognostic biomarker or a predictive marker for treatment response or relapse.

## Abbreviations

3D: 3-dimensional; FFPE: formalin fixed paraffin embedded; HGF: hepatocyte growth factor; MTT: methylthiazolyldiphenyl-tetrazolium; VSVG: vesicular stomatitis virus.

## Competing interests

The authors declare that they have no competing interests.

## Authors' contributions

HM (MSc student with BEE) performed western blotting, wound healing, tumor engraftment experiments and data analysis. AN (PhD student with MA) generated tumor cell clones expressing the Y477F ezrin mutant, and assisted in interpretation of immunofluorescence and metastasis results. SV (Pathology resident with SS) set up criteria by which to assess gross and microscopic pathology of primary and metastatic lesions, and provided histopathology expertise. AD performed statistical analyses. CS carried out the 3D culture studies and assisted in animal experimentation. SS was the overall pathology consultant on the project. MA provided the initial concept, and the ezrin mutant constructs used in the study. BEE provided expertise in cell function assays and tumor engraftment models. All authors read and approved the final manuscript.

## Pre-publication history

The pre-publication history for this paper can be accessed here:

http://www.biomedcentral.com/1471-2407/12/82/prepub

## Supplementary Material

Additional file 1 **Figure S1**. Effect of Y477F ezrin on growth of AC2M2 cells in 3D Matrigel cultures. Panels A and B) AC2M2 cell clones expressing pCB6 empty vector or Y477F ezrin (clones A43 and C13) were cultured in 3D Matrigel. Representative phase contrast images of 9 day cultures of clones pCB6 (**A**) and C13 (**B**) photographed with a 4x objective are shown. **Panel C) **AC2M2 cell clones described above were cultured in 96 well plates (104 cells/100 μl/well) with 20% Matrigel, supplemented with Phenol Red-free complete DMEM medium. After 3 days, an MTT assay was performed according to the manufacturer's instructions. Values represent mean O.D. (Absorbance at 570 nm) of 4 wells +/- SD. No significant difference in growth was detected as determined by one way ANOVA (*p *= 0.319). **Panel D) **The number of colonies per well in 9 day cultures described in A and B was counted visually using phase contrast microscopy, and plotted as the mean of three wells +/- SD. The colony forming ability (% colonies per 7.5 x 103 cells plated) for each group is indicated. A marginal increase in colony forming ability was apparent in A43 and C13 (perhaps due to some clustering of more diffuse pCB6 colonies), but this difference was not significant as determined by one way ANOVA. Click here for file

Additional file 2 **Figure S2**. Effect of the expression of Y477F ezrin on primary tumor growth: Mice injected in the mammary fat pad with AC2M2 cell clones (pCB6, A43, C13) in Figure 2 were monitored every two days, and palpable tumors were measured using Vernier calipers. Primary tumors were excised after 21-23 days, and mice were allowed to live for 40 days (20 days post-resection of the primary tumor). **Panel A) **Equal protein amounts of primary tumor tissue lysates were subjected to SDS-PAGE. Western blotting was performed using antibodies against VSVG, ezrin and γ-tubulin. Mean optical density ratios of ezrin vs γtubulin bands for the three tumor groups (0.52, 0.55 and 0. 64) showed no significant difference, most likely due to host tissue contribution to the total ezrin pool. **Panel B) **Primary tumor volumes in each group were plotted as a function of days post engraftment. The mean +/- one standard deviation (bars) is indicated for each group (**pCB6, A43, C13**) at each time point. The natural log of the tumor volumes as measured on days 15, 17, 20, and 23 was compared among the groups by a linear mixed effect model as estimated by restricted maximum likelihood using the SAS MIXED procedure (SAS Institute Inc., 2008). A first order autoregressive correlation structure was used to account for within mouse dependence [39]. No significance among groups on all four days was detected, using a global F-test (overall *p *= 0.57). Click here for file

Additional file 3 **Figure S3**. Histopathology of local invasion and metastasis of pCB6 tumors. Representative examples of local invasion and distant metastasis from mammary tumors expressing empty pCB6 vector in experiments from Figures 4 and 5 are shown. Primary tumors were excised after 21-23 days, and animals were allowed to survive for a total of 40 days. Primary tumors (**Panels A-C**), excised previously, and additional organs (**Panels D-H**) were retrieved by detailed autopsy and were processed for histopathological analyses. FFPE processed tissues were sectioned (5 μm), and stained with hematoxylin and eosin. Images show the diverse invasive and metastatic characteristics of the pCB6 control tumors, including a suggestion of perineural invasion (**Panels A,B**), direct invasion into the fatpad (**Panel C**), seeding of the small intestine mesentery and serosal invasion (**Panel D**), pancreatic invasion (**Panel E)**, seeding of the splenic capsule (**Panel F**), and lung metastases (**Panels G and H**). Label with "T" indicates primary tumor, "N" indicates nerve encased by tumor cells, "M" indicates mucosa, "S" indicates serosa, "P" indicates pancreatic acini, "SC" indicates splenic capsule, and "met" indicates metastatic nodule. Image magnifications are indicated in lower right corner of each image. Click here for file
