# Supplementary figures and images for: Ezrin phosphorylation on tyrosine 477 regulates invasion and metastasis of breast cancer cells
Source: BMC Cancer. 2012 Mar 7;12:82. doi: 10.1186/1471-2407-12-82 (PMC3372425; doi:10.1186/1471-2407-12-82)

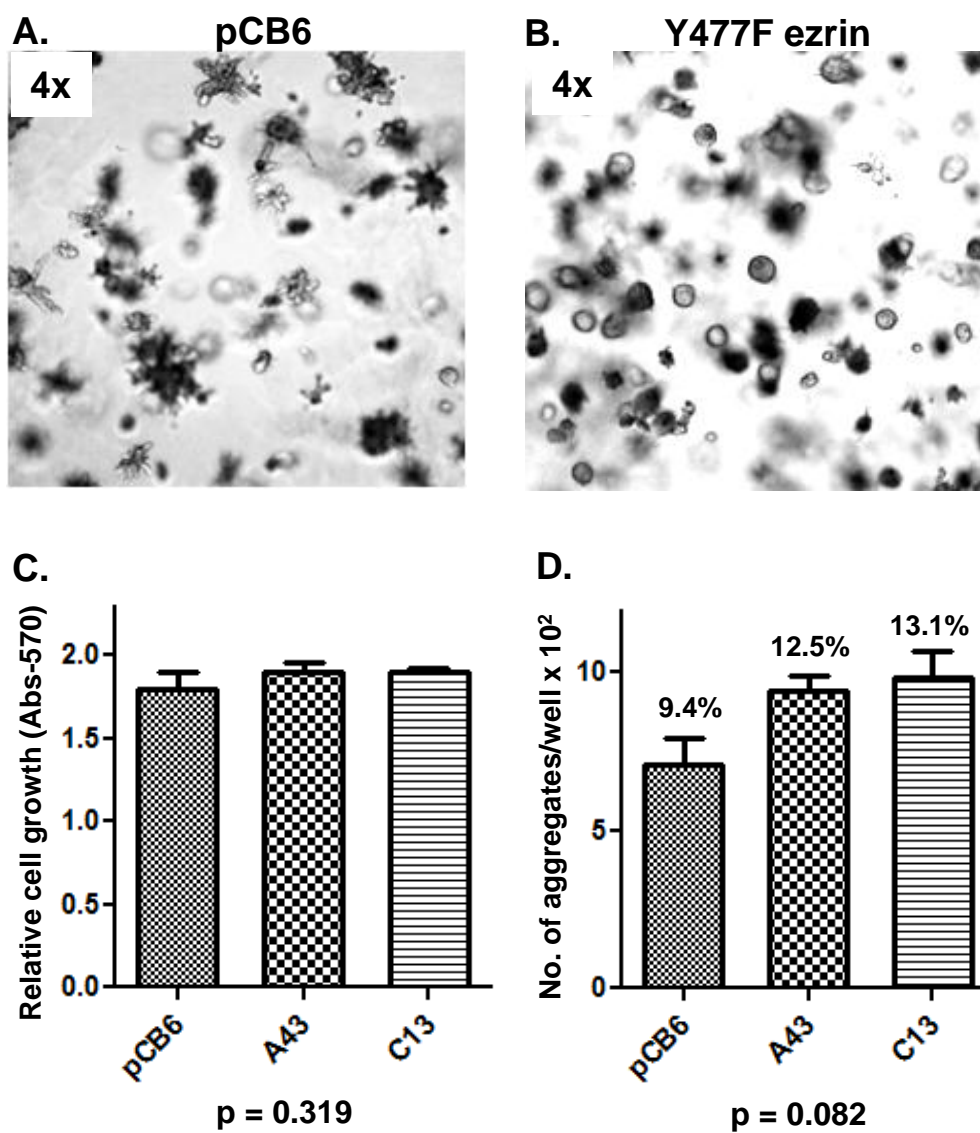

Supplement: Additional file 1 — Figure S1. Effect of Y477F ezrin on growth of AC2M2 cells in 3D Matrigel cultures. Panels A and B) AC2M2 cell clones expressing pCB6 empty vector or Y477F ezrin (clones A43 and C13) were cultured in 3D Matrigel. Representative phase contrast images of 9 day cultures of clones pCB6 (A) and C13 (B) photographed with a 4x objective are shown. Panel C) AC2M2 cell clones described above were cultured in 96 well plates (104 cells/100 μl/well) with 20% Matrigel, supplemented with Phenol Red-free complete DMEM medium. After 3 days, an MTT assay was performed according to the manufacturer's instructions. Values represent mean O.D. (Absorbance at 570 nm) of 4 wells +/- SD. No significant difference in growth was detected as determined by one way ANOVA (p = 0.319). Panel D) The number of colonies per well in 9 day cultures described in A and B was counted visually using phase contrast microscopy, and plotted as the mean of three wells +/- SD. The colony forming ability (% colonies per 7.5 x 10^3^ cells plated) for each group is indicated. A marginal increase in colony forming ability was apparent in A43 and C13 (perhaps due to some clustering of more diffuse pCB6 colonies), but this difference was not significant as determined by one way ANOVA. [file 1471-2407-12-82-S1.PDF]

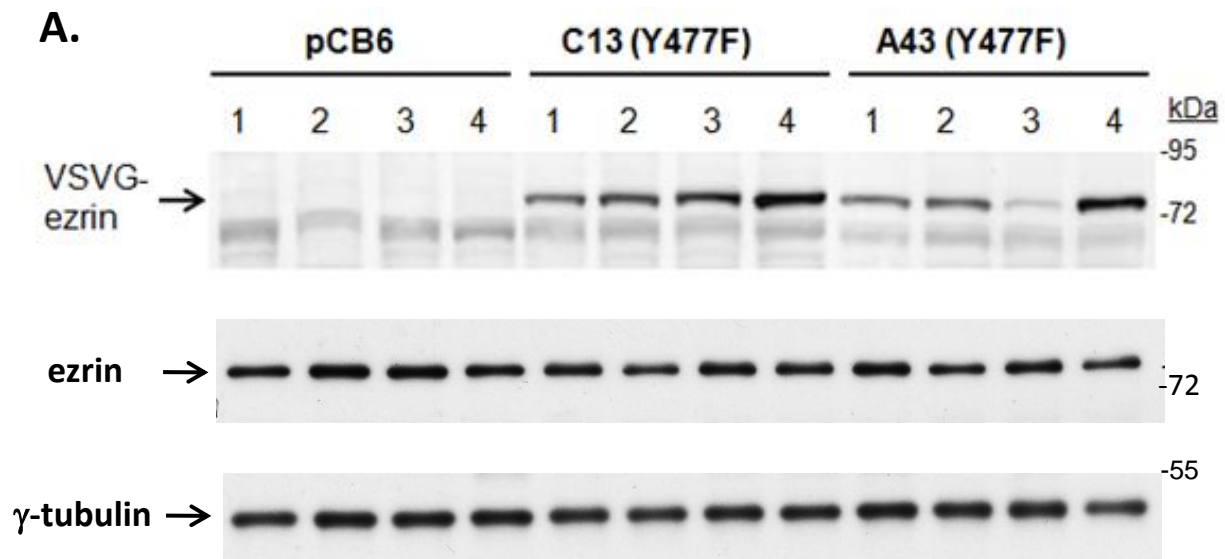

**B.**

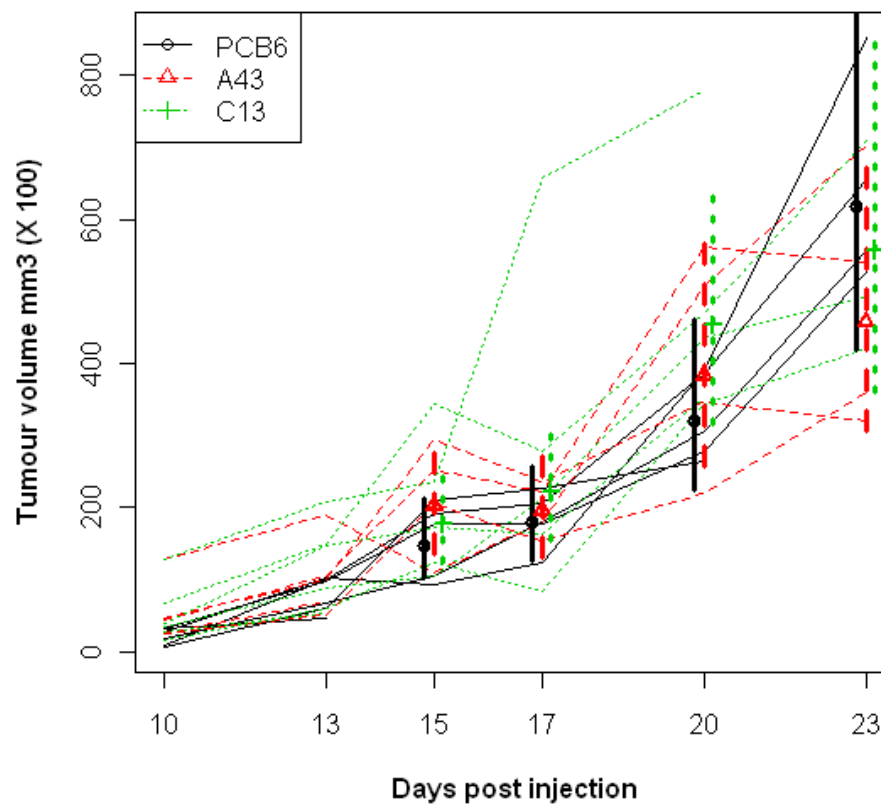

Supplement: Additional file 2 — Figure S2. Effect of the expression of Y477F ezrin on primary tumor growth: Mice injected in the mammary fat pad with AC2M2 cell clones (pCB6, A43, C13) in Figure 2 were monitored every two days, and palpable tumors were measured using Vernier calipers. Primary tumors were excised after 21-23 days, and mice were allowed to live for 40 days (20 days post-resection of the primary tumor). Panel A) Equal protein amounts of primary tumor tissue lysates were subjected to SDS-PAGE. Western blotting was performed using antibodies against VSVG, ezrin and γ-tubulin. Mean optical density ratios of ezrin vs γtubulin bands for the three tumor groups (0.52, 0.55 and 0. 64) showed no significant difference, most likely due to host tissue contribution to the total ezrin pool. Panel B) Primary tumor volumes in each group were plotted as a function of days post engraftment. The mean +/- one standard deviation (bars) is indicated for each group (pCB6, A43, C13) at each time point. The natural log of the tumor volumes as measured on days 15, 17, 20, and 23 was compared among the groups by a linear mixed effect model as estimated by restricted maximum likelihood using the SAS MIXED procedure (SAS Institute Inc., 2008). A first order autoregressive correlation structure was used to account for within mouse dependence [39]. No significance among groups on all four days was detected, using a global F-test (overall p = 0.57). [file 1471-2407-12-82-S2.PDF]
