# Supplementary material for: Ezrin phosphorylation on tyrosine 477 regulates invasion and metastasis of breast cancer cells
Source: BMC Cancer. 2012 Mar 7;12:82. doi: 10.1186/1471-2407-12-82 (PMC3372425; doi:10.1186/1471-2407-12-82)

Additional Figure 3 file: Figure S3 Mak et al.

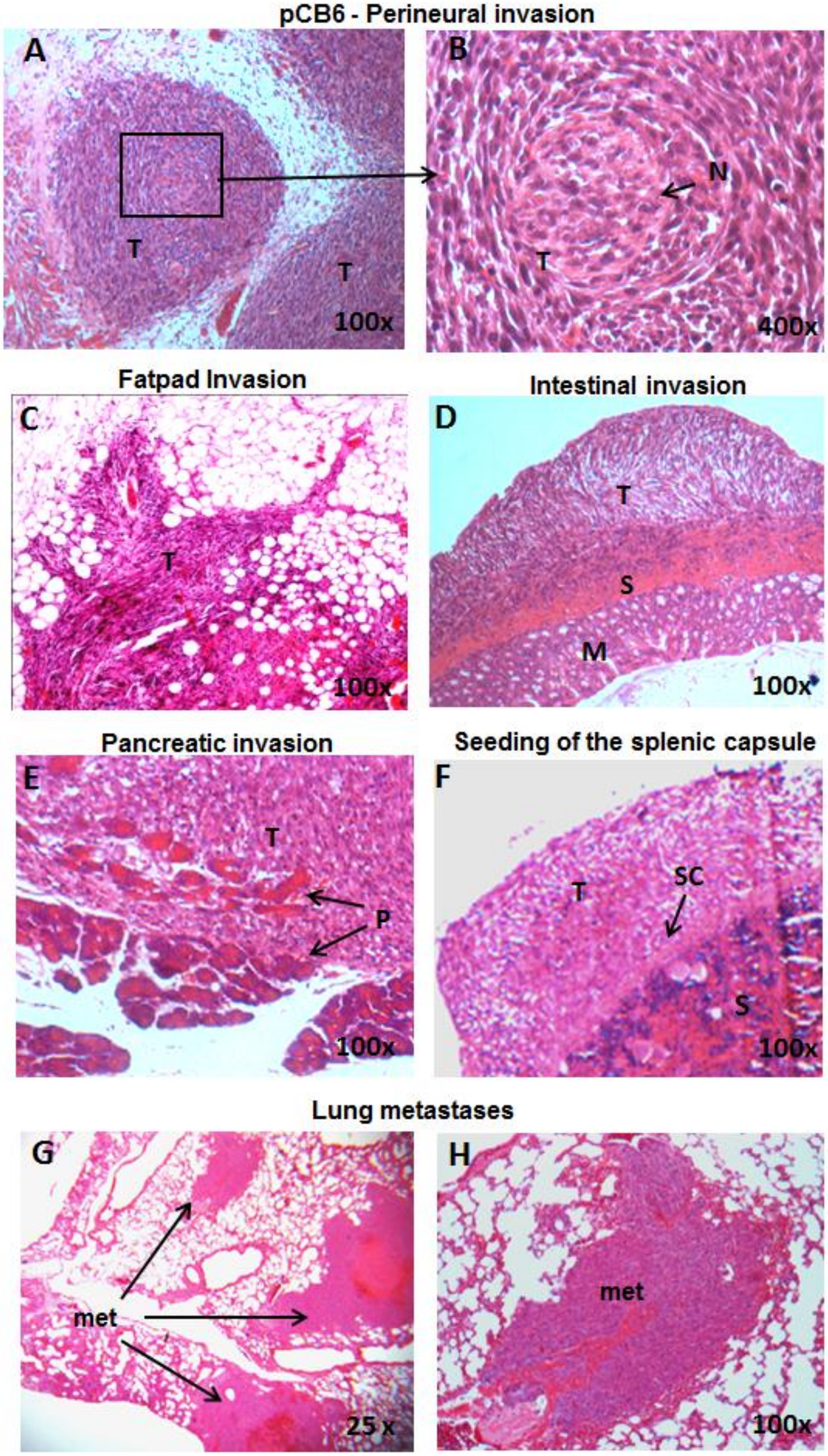

Supplement: Additional file 3 — Figure S3. Histopathology of local invasion and metastasis of pCB6 tumors. Representative examples of local invasion and distant metastasis from mammary tumors expressing empty pCB6 vector in experiments from Figures 4 and 5 are shown. Primary tumors were excised after 21-23 days, and animals were allowed to survive for a total of 40 days. Primary tumors (Panels A-C), excised previously, and additional organs (Panels D-H) were retrieved by detailed autopsy and were processed for histopathological analyses. FFPE processed tissues were sectioned (5 μm), and stained with hematoxylin and eosin. Images show the diverse invasive and metastatic characteristics of the pCB6 control tumors, including a suggestion of perineural invasion (Panels A,B), direct invasion into the fatpad (Panel C), seeding of the small intestine mesentery and serosal invasion (Panel D), pancreatic invasion (Panel E), seeding of the splenic capsule (Panel F), and lung metastases (Panels G and H). Label with "T" indicates primary tumor, "N" indicates nerve encased by tumor cells, "M" indicates mucosa, "S" indicates serosa, "P" indicates pancreatic acini, "SC" indicates splenic capsule, and "met" indicates metastatic nodule. Image magnifications are indicated in lower right corner of each image. [file 1471-2407-12-82-S3.PDF]
